# RGB-D SLAM Based on Extended Bundle Adjustment with 2D and 3D Information

**DOI:** 10.3390/s16081285

**Published:** 2016-08-13

**Authors:** Kaichang Di, Qiang Zhao, Wenhui Wan, Yexin Wang, Yunjun Gao

**Affiliations:** 1 State Key Laboratory of Remote Sensing Science, Institute of Remote Sensing and Digital Earth, Chinese Academy of Sciences, No. 20A, Datun Road, Chaoyang District, Beijing 100101, China; dikc@radi.ac.cn (K.D.); zhaoqiang@radi.ac.cn (Q.Z.); wangyx716@radi.ac.cn (Y.W.); gaoyunjunsx@163.com (Y.G.); 2 University of Chinese Academy of Sciences, Beijing 100049, China

**Keywords:** RGB-D camera, SLAM, projection model, bundle adjustment, Kinect

## Abstract

In the study of SLAM problem using an RGB-D camera, depth information and visual information as two types of primary measurement data are rarely tightly coupled during refinement of camera pose estimation. In this paper, a new method of RGB-D camera SLAM is proposed based on extended bundle adjustment with integrated 2D and 3D information on the basis of a new projection model. First, the geometric relationship between the image plane coordinates and the depth values is constructed through RGB-D camera calibration. Then, 2D and 3D feature points are automatically extracted and matched between consecutive frames to build a continuous image network. Finally, extended bundle adjustment based on the new projection model, which takes both image and depth measurements into consideration, is applied to the image network for high-precision pose estimation. Field experiments show that the proposed method has a notably better performance than the traditional method, and the experimental results demonstrate the effectiveness of the proposed method in improving localization accuracy.

## 1. Introduction

Simultaneous localization and mapping (SLAM) is the process of incrementally estimating the pose of a moving platform and generating the surrounding map from the apparent motion induced on the images of its onboard cameras, and is considered to be a key prerequisite of truly autonomous robots [1,2,3,4]. This capability of simultaneously localizing a robot and accurately mapping its environment makes it vitally important, especially in GPS-denied environments such as lunar and Martian surface. Vision-based SLAM has been successfully applied to planetary exploration missions, such as the NASA’s Mars Exploration Rover 2003 (MER) mission [5,6,7] and China’s Chang’E-3 mission [8]. In these applications, vision-based localization method can effectively reduce error accumulation caused by wheel slippage and/or inertial measurement unit (IMU) drift. Visual SLAM is usually realized by stereo camera, and 3D information of the traversing area is obtained by dense matching. This usually requires a large amount of computation time and will reduce the efficiency of rover traversing and exploration. In addition, in case of insufficient image texture due to the natural environment or illumination condition, visual localization may become inaccurate or completely fail.

RGB-D camera is a new type of sensor which can provide both visual texture information and per-pixel depth information simultaneously. Regardless of texture and illumination condition, a RGB-D camera can directly obtain 3D information in the scene with the depth camera using active imaging mode. Therefore, RGB-D camera has a natural advantage for spatial information acquisition in restricted environments such as lunar or Martian surface. In recent years, low-cost and real-time RGB-D sensors, such as Microsoft Kinect (V1 and V2), Intel RealSense, and Leap Motion, have been applied in motion sensing games, human-computer interaction, and other areas. Due to the low-cost and real-time nature of the sensor, RGB-D cameras have become a hotspot for 3D applications. Microsoft’s KinectFusion system [9,10] enables a user holding and moving a standard Kinect camera to rapidly create detailed 3D reconstructions of an indoor scene. Khoshelham and Elberink [11] described the principle of obtaining depth image and RGB image of Kinect V1, and did 3D reconstruction of objects based on depth data. Smisek et al. [12] and Daniel et al. [13] presented a calibration method of Kinect and gave the error characteristics of the depth camera and the RGB camera of Kinect V1. Butkiewicz et al. [14] and Fankhauser et al. [15] have done comprehensive analyses of the error characteristics of the depth camera of Kinect V2. Lee and Ho [16] and Chen et al. [17] presented several methods to eliminate the noise of depth data based on bilateral filtering, median filtering, and 3D curvature analysis.

In recent years, many researches about visual SLAM with RGB-D camera have been reported. One of the earliest published RGB-D SLAM system was proposed by Henry et al. [18], in which visual features are used in combination with generalized iterative closest point (ICP) algorithm to create and optimize a pose graph. Huang et al. [19] developed a RGB-D SLAM method in which sparse bundle adjustment (SBA) is used for global consistency by minimizing the matching errors of the visual FAST feature correspondences between frames. The similar method was adopted with visual feature correspondences, which were used in conjunction with pose graph optimization [20,21]. In [22,23], depth measurement is utilized as constraints into bundle adjustment in which error function is established by transforming the landmarks in the current frame back into the previous frame and minimizing 3D alignment error in the two frames, then loop closure is detected and utilized to improve the accuracy and robustness. Dryanovski et al. [24] realized indoor SLAM using Kalman Filter and loop closure detection to optimize the camera pose estimation obtained by ICP algorithm. Whelan et al. [25] presented the method of large scale dense RGB-D SLAM based on volumetric fusion and truncated signed distance function (TSDF), which broke through the scope limitation of KinectFusion and made optimization in loop closure detection. Heredia et al. [26] improved the speed and robustness of localization by feature matching exclusively in high-dimensional feature space. A novel approach based on Kalman prediction and filtering with intermittent observations identified from depth image segmentation was proposed in [27].

In planetary exploration, a rover usually moves from a science object to the next, and usually will not go back to the previous place where it has explored. Thus, it is impossible to use loop closure detection to optimize the pose graph. In addition, the complex conditions of illumination and surface texture may make the traditional methods, which only depend on visual feature tracking, infeasible.

In this paper, based on the measurement capability of the RGB-D camera system, an extended bundle adjustment (BA) based SLAM method with integrated 2D and 3D measurements from Kinect is presented. Unlike the traditional BA method [19,20,21] used in RGB-D camera SLAM, which refines the camera pose estimation using the projection model and error model that only constructed with visual information, depth information is not only used to generate 3D scene, but also introduced into the BA model as one type of the primary measurement data in our method. Compared with the BA method [22,23] which introduced the depth measurements as 3D constraints into the BA model, our method takes depth measurements as independent observations and integrates them with image measurements through the projection model and error model. In this way, the error characteristics of the depth measurements can be taken into consideration by the error model, and would result in better result of pose estimation. Results of field experiments are given to verify the accuracy and effectiveness of this new method.

## 2. Methodology

**Figure 1:**
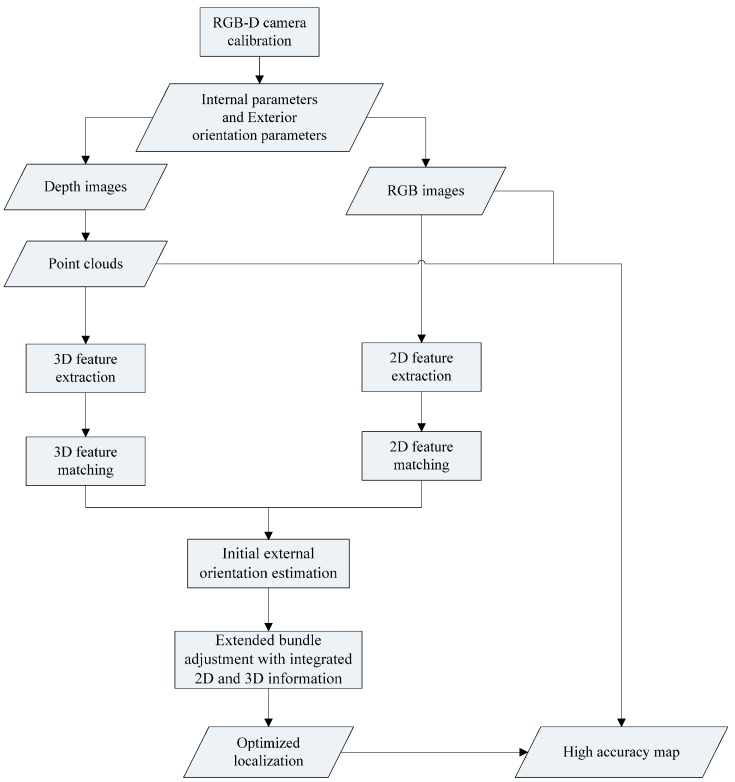
Flowchart of our method.

The flowchart of our method is shown in Figure 1, which consists of the following steps: (1) RGB image and depth image registration; (2) 2D and 3D feature detection and matching; (3) initial exterior orientation calculation; (4) high-accuracy exterior orientation estimation by extended bundle adjustment with 2D and 3D information.

### 2.1. RGB Image and Depth Image Registration through Camera Calibration

The RGB image and the simultaneously acquired depth image should be registered first in order to use them in an integrated way in the SLAM process. Although a function is given in the Microsoft Kinect SDK to register the RGB camera and depth camera, errors of several pixels still exist in the registration results. Therefore it is necessary to calibrate the RGB-D camera before it can be used for accurate measurement.

The Kinect depth camera is actually an IR camera, which obeys the principle of pinhole imaging. Traditional camera calibration model, which contains lens distortion coefficients [*k*_1_, *k*_2_, *k*_3_, *p*_1_, *p*_2_], is adopted for both the RGB camera and the depth camera. Image coordinate system o-xy is defined such that origin is the center of the lower left pixel of the image, the x axis is horizontal to the right, and y axis is vertical up. The lens distortion model can be represented by Equation (1):

(1) $$\{\begin{array}{c} x^{d}=x+\delta_{x},y^{d}=y+\delta_{y},r^{2}=x^{2}+y^{2}\\ \left(\begin{array}{c} \delta_{x}\\ \delta_{y} \end{array}\right)=\left(\begin{array}{c} x(k_{1}r^{2}+k_{2}r^{4}+k_{3}r^{6})\\ y(k_{1}r^{2}+k_{2}r^{4}+k_{3}r^{6}) \end{array}\right)+\left(\begin{array}{c} 2p_{1}xy+p_{2}(r^{2}+2x^{2})\\ p_{1}(r^{2}+2y^{2})+2p_{1}xy \end{array}\right) \end{array}$$

where (*δ_x_*, *δ_y_*) is the camera distortion along x direction and y direction, (*x^d^*,*y^d^*) is the original image coordinates of an image point, (*x*,*y*) is the image coordinates after distortion correction.

**Figure 2:**
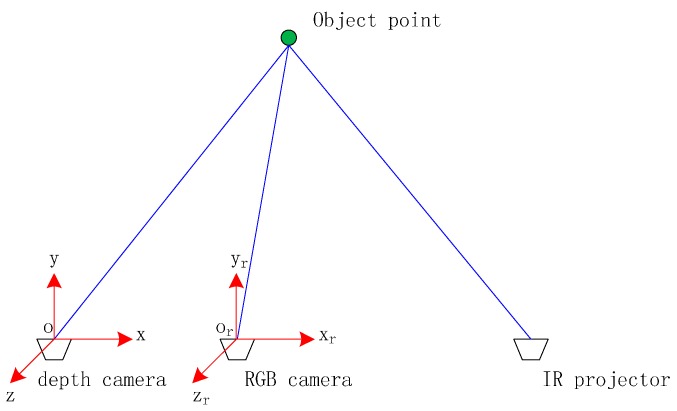
Spatial relationship of Kinect cameras.

Figure 2 shows the spatial relationship between the depth camera (IR camera) and the RGB camera. o-xyz and o_r_-x_r_y_r_z_r_ are defined as the coordinate systems of the depth camera and the RGB camera, respectively, whose origins coincide with their respective camera optical centers. The z (z_r_) axis points along the optical axis, the x (x_r_) axis is horizontal to the right and perpendicular to z (z_r_) axis, the y (y_r_) axis is defined to form a right-handed system. The transformation relationship between the RGB camera and the depth camera can be expressed by a 3 × 3 rotation matrix **R** and a translation vector $T={\left[X_{S},Y_{S},Z_{S}\right]}^{T}$. Supposing that there is an object point (X,Y,Z), the depth value obtained by depth camera is *d* and the projected points on the depth image and the RGB image are $\left(x_{D},y_{D}\right)$ and $\left(x_{R},y_{R}\right)$, respectively. Take the depth camera coordinate system as the reference coordinate system, the imaging geometric models of the two cameras can be represented as:

(2) $$\left\{ \begin{array}{l} X=\frac{(x_{D}-x_{0D})\cdot d}{f_{Dx}}\\ Y=\frac{(y_{D}-y_{0D})\cdot d}{f_{Dy}}\\ Z=-d \end{array}\right.$$

(3) $$\left\{ \begin{array}{l} x_{R}-x_{0R}=-f_{Rx}\frac{R_{11}(X-X_{S})+R_{12}(Y-Y_{S})+R_{13}(Z-Z_{S})}{R_{31}(X-X_{S})+R_{32}(Y-Y_{S})+R_{33}(Z-Z_{S})}\\ y_{R}-y_{0R}=-f_{Ry}\frac{R_{21}(X-X_{S})+R_{22}(Y-Y_{S})+R_{23}(Z-Z_{S})}{R_{31}(X-X_{S})+R_{32}(Y-Y_{S})+R_{33}(Z-Z_{S})} \end{array}\right.$$

where $f_{D}=\left[f_{Dx},f_{Dy}\right]$,$f_{R}=\left[f_{Rx},f_{Ry}\right]$, $\left(x_{0D},y_{0D}\right)$ and $\left(x_{0R},y_{0R}\right)$ are the focal lengths and principal points of the depth camera and the RGB camera, *R_ij_* (*i, j* = 1, 2, 3) are the elements of the rotation matrix R of the RGB camera with respect to the depth camera. Once the rotation matrix R and the translation vector T are determined through camera calibration based on Equations (1)–(3), the registration between the simultaneously acquired depth image and RGB image can be easily achieved.

In our research, the camera calibration method proposed by Smisek [12] is adopted. The Kinect cameras are calibrated together using a planer checkerboard by blocking the IR projector and illuminating the target checkerboard by an infrared lamp. Camera Calibration Toolbox for Matlab is used to complete the calibration with the images taken from different distances and orientations. Finally, the camera internal (interior) parameters, lens distortion coefficients, rotation matrix, and translation vector are obtained through the calibration.

The calibration result is shown in Table 1 and Table 2. The calibration accuracy can be depicted by the residual error in image space. As a result, the standard deviations of the image residuals are less than 0.3 pixel for both the RGB camera and the depth camera. As the relative geometric parameters are obtained through RGB-D camera calibration, every pixel in the depth image can be rendered with certain RGB value of the corresponding pixel in the RGB image, and colored 3D point clouds are generated from the registered RGB-D image.

### 2.2. 2D and 3D Feature Detection and Matching

The texture data containing 2D visual features of the scene in gray scale and the depth data containing 3D feature of the scene, are two types of data obtained simultaneously by a RGB-D camera. Taking full advantage of both of the two types of data can obtain more features and improve the localization accuracy.

SIFT feature [28] is adopted in our approach to extract 2D visual features in the registered image. A GPU based implementation of SIFT [29] is used to speed up the process of keypoint detection and descriptor computation. Matching of extracted keypoints in consecutive frames is followed by the Random Sample Consensus (RANSAC) algorithm implementation, which is used to eliminate outliers from the matched results. The inlier features’ locations are projected from the registered image to 3D correspondences (as described below) using Equations (2) and (3). It should be noted that the coordinates of the matched keypoints in the image are not integers, so the depth value of a keypoint is calculated through bilinear interpolation.

**Figure 3:**
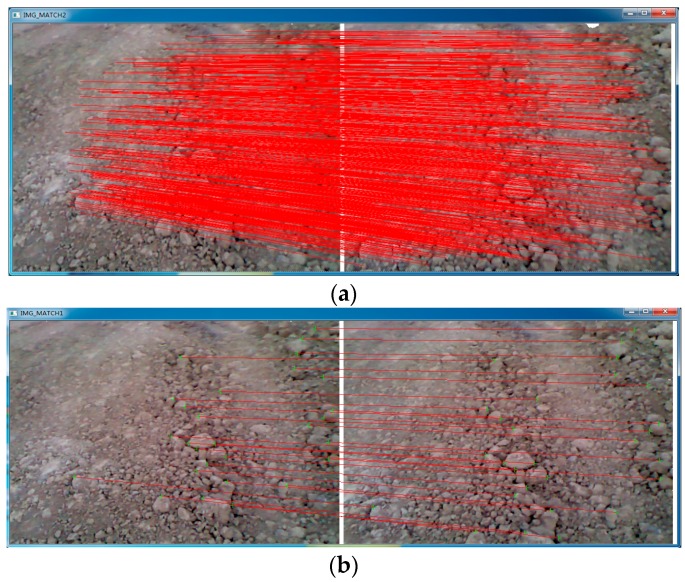
Features detection and matching results. (**a**) 2D feature detection and matching; (**b**) 3D feature detection and matching.

3D feature, which represents the spatial geometric attribution, is utilized in our research. Based on the point clouds derived from the registered image, Normal Aligned Radial Feature (NARF) is used to extract interest points and Fast Point Feature Histograms (FPFH) are applied to compute descriptors in this paper. NARF interest point extraction method (as introduced in [30]) operates on range images generated from arbitrary 3D point clouds and considers the borders of the objects identified by transitions from foreground to background. After extracting keypoints, feature descriptors should be computed for each keypoint in order to compare with the corresponding descriptors to find corresponding points in different point clouds of the same scene. Fast Point Feature Histograms (FPFH) [31] are used in our method, because FPFH is fast to compute, relatively stable, and leads to superior results compared with other descriptors [32]. As a result, all the matched keypoints have both 2D and 3D data. The result of features detection and matching is shown in Figure 3.

2D features are detected in the registered images and 3D features are re-projected to the registered images, so that all the 2D features and 3D features have the image plane coordinates values and the depth measurement values. These matched feature points are used as tie points to link consecutive frames to build a continuous image network. In consideration of accuracy and efficiency of bundle adjustment of the image network, only the object points which have projections in two to five images are used. Mean coordinate values of object points in all projected frames are calculated as initial values in BA solution.

### 2.3. Initial Exterior Orientation Calculation

The goal of this step is to calculate initial exterior orientation parameters of each frame. Based on the 2D and 3D features, rigid transformation is performed to estimate the camera pose of every frame with respect to its previous frame, and the transformation model can be described as follows:

(4) $$d_{i}=Rm_{i}+T+V_{i}$$

where **R** is a standard 3 × 3 rotation matrix, **T** is a 3 × 1 translation vector and **V***_i_* is a 3 × 1 error vector [33]. Solving the optimal transformation [R,T] that maps the feature points set {*m_i_*} in the first frame onto the feature points set {*d_i_*} in the next frame requires a least squares calculation, as follows:

(5) $$\sum V_{i}{}^{2}=\sum_{i=1}^{n}||d_{i}-Rm_{i}-T|{|}^{2}$$

In this paper, singular value decomposition (SVD) method [34] is adopted to minimize Equation (5), so that the initial exterior orientation [R,T] can be obtained.

### 2.4. Extended Bundle Adjustment with Image and Depth Measurements

Initial camera pose of every frame is calculated in the above section, but inevitable drift caused by measurement errors, feature point matching errors, and so on, will accumulate rapidly over space and time. Bundle adjustment of the image network—which is the technique of refining a visual reconstruction to produce jointly optimal 3D structure and orientation parameters estimated by using accurate projection model, statistical error models, and well-developed quality control methodology [35,36]—is used to optimize the initial exterior orientation result. Constructing the projection model and error model of RGB-D camera is the key to achieve optimal estimation. Many approaches based on BA have been proposed to reduce drift [19,20,21,37] refining camera poses. However, in these methods, projection models and error models are built only considering the visual measurement information, depth information which is only used to calculate the 3D coordinate is not brought into BA model as another primary measurement data, which means that these BA models are not fully utilizing the measurement capability provided by the RGB-D camera system. In our method, we present a new projection model of RGB-D camera using the two types of primary measurement data and build an accurate error model based on the projection model.

#### 2.4.1. Projection Model

**Figure 4:**
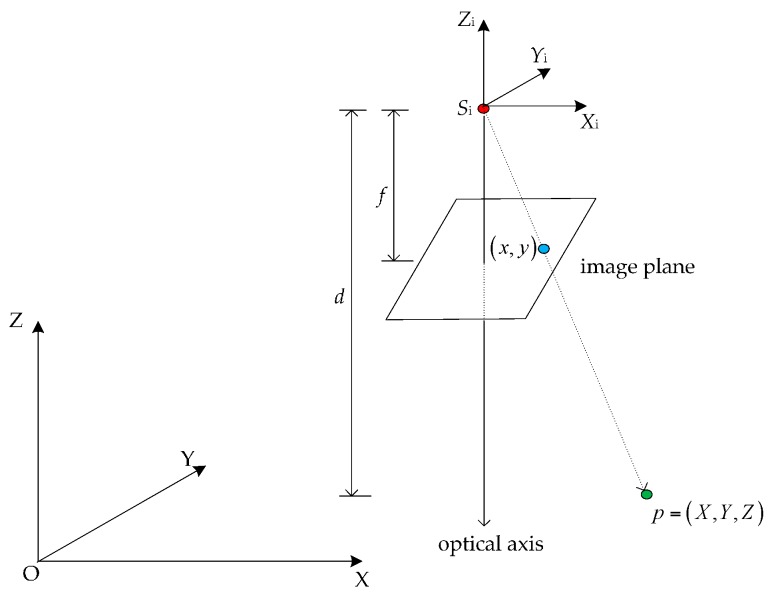
Illustration of projection model of RGB-D camera.

The projection model of a RGB-D camera represents the relationship of an object point in the real world and its measurements in the RGB-D images. There are two types of measurements: image coordinates from the RGB-image and depth values from the depth image. As shown in Figure 4, supposing the position of the depth camera is *S*_i_ in the world coordinate system O-XYZ. The camera pose is R and $T=\left[X_{S},Y_{S},Z_{S}\right]$ which express the relationship between the world coordinate system and the local camera coordinate system S_i_-X_i_Y_i_Z_i_. For an object point p=(X,Y,Z), its image plane coordinates is (*x*,*y*) and the depth value is *d*.

The collinearity equation model can be expressed as:

(6) $$\left[\begin{array}{l} X-X_{S}\\ Y-Y_{S}\\ Z-Z_{S} \end{array}\right]=\lambda R\left[\begin{array}{l} x\\ y\\ -f \end{array}\right]$$

for a RGB-D camera, Equation (6) can be rewritten as:

(7) $$\left[\begin{array}{l} X-Z_{S}\\ Y-Y_{S}\\ Z-Z_{S} \end{array}\right]=\left(\begin{array}{ccc} a_{1} & a_{2} & a_{3}\\ b_{1} & b_{2} & b_{3}\\ c_{1} & c_{2} & c_{3} \end{array}\right)\left[\begin{array}{l} \lambda x\\ \lambda y\\ -d \end{array}\right]$$

then the following equation is obtained:

(8) $$\left\{ \begin{array}{l} x-x_{0}=-f_{x}\frac{a_{1}(X-X_{S})+b_{1}(Y-Y_{S})+c_{1}(Z-Z_{S})}{a_{3}(X-X_{S})+b_{3}(Y-Y_{S})+c_{3}(Z-Z_{S})}\\ y-y_{0}=-f_{y}\frac{a_{2}(X-X_{S})+b_{2}(Y-Y_{S})+c_{2}(Z-Z_{S})}{a_{3}(X-X_{S})+b_{3}(Y-Y_{S})+c_{3}(Z-Z_{S})}\\ \;\\ d=-[a_{3}(X-X_{S})+b_{3}(Y-Y_{S})+c_{3}(Z-Z_{S}\text{)]} \end{array}\right.$$

where $\left(x_{0},y_{0}\right)$is the principal point and $f=\left[f_{x},f_{y}\right]$is the focal length of the depth camera. Equation (8) is the projection model of the RGB-D camera.

#### 2.4.2. Error Model

In the projection model, the relationship between the measurement data and the unknowns is nonlinear. To simplify the solution, it is necessary to linearize Equation (8) by Taylor series expansion with the constant terms and first-degree terms remained. The linearized equation can be represented as:

(9) $$\left\{ \begin{array}{l} v_{x}=(x)-x+\frac{\partial x}{\partial X_{s}}\Delta X_{s}+\frac{\partial x}{\partial Y_{s}}\Delta Y_{s}+\frac{\partial x}{\partial Z_{s}}\Delta Z_{s}+\frac{\partial x}{\partial \omega }\Delta \omega +\frac{\partial x}{\partial \phi }\Delta \phi +\frac{\partial x}{\partial \kappa }\Delta \kappa +\frac{\partial x}{\partial X}\Delta X+\frac{\partial x}{\partial Y}\Delta Y+\frac{\partial x}{\partial Z}\Delta Z\\ v_{y}=(y)-y+\frac{\partial y}{\partial X_{s}}\Delta X_{s}+\frac{\partial y}{\partial Y_{s}}\Delta Y_{s}+\frac{\partial y}{\partial Z_{s}}\Delta Z_{s}+\frac{\partial y}{\partial \omega }\Delta \omega +\frac{\partial y}{\partial \phi }\Delta \phi +\frac{\partial y}{\partial \kappa }\Delta \kappa +\frac{\partial y}{\partial X}\Delta X+\frac{\partial y}{\partial Y}\Delta Y+\frac{\partial y}{\partial Z}\Delta Z\\ v_{d}=(d)-d+\frac{\partial d}{\partial X_{s}}\Delta X_{s}+\frac{\partial d}{\partial Y_{s}}\Delta Y_{s}+\frac{\partial d}{\partial Z_{s}}\Delta Z_{s}+\frac{\partial d}{\partial \omega }\Delta \omega +\frac{\partial d}{\partial \phi }\Delta \phi +\frac{\partial d}{\partial \kappa }\Delta \kappa +\frac{\partial d}{\partial X}\Delta X+\frac{\partial d}{\partial Y}\Delta Y+\frac{\partial d}{\partial Z}\Delta Z \end{array}\right.$$

where (*x*), (*y*), and (*z*) are the constant terms which can be calculated in Equation (6); (ω,ϕ,κ)are the attitude angles. A series of parameters are used to simplify the expression of Equation (9):

(10) $$\left\{ \begin{array}{l} v_{x}=a_{11}\Delta X_{s}+a_{12}\Delta Y_{s}+a_{13}\Delta Z_{s}+a_{14}\Delta \omega +a_{15}\Delta \phi +a_{16}\Delta \kappa +a_{17}\Delta X+a_{18}\Delta Y+a_{19}\Delta Z-l_{x}\\ v_{y}=a_{21}\Delta X_{s}+a_{22}\Delta Y_{s}+a_{23}\Delta Z_{s}+a_{24}\Delta \omega +a_{25}\Delta \phi +a_{26}\Delta \kappa +a_{27}\Delta X+a_{28}\Delta Y+a_{29}\Delta Z-l_{y}\\ \;\\ v_{d}=a_{31}\Delta X_{s}+a_{32}\Delta Y_{s}+a_{33}\Delta Z_{s}+a_{34}\Delta \omega +a_{35}\Delta \phi +a_{36}\Delta \kappa +a_{37}\Delta X+a_{38}\Delta Y+a_{39}\Delta Z-l_{d} \end{array}\right.$$

The method of deriving the coefficients (partial derivatives) of *v_x_* and *v_y_* in the equation is described in detail by Wang [36]. In this paper, we only elaborate the process of deriving the coefficients in the equation *v_d_*. Using the attitude angles the depth value can be expressed in another way:

(11) $$d=-\sin\phi (X-X_{S})+\sin\omega \cos\phi (Y-Y_{S})-\cos\omega \cos\phi (Z-Z_{S})$$

So we can get the partial derivatives as follows:

$$\left\{ \begin{array}{l} a_{31}=\frac{\partial d}{\partial X_{s}}=\sin\phi \;\;\;a_{32}=\frac{\partial d}{\partial Y_{s}}=-\sin\omega \cos\phi \;\;\;a_{33}=\frac{\partial d}{\partial Z_{s}}=\cos\omega \cos\phi \\ a_{34}=\frac{\partial d}{\partial \omega }=\cos\omega \cos\phi (Y-Y_{s})+\sin\omega \cos\phi (Z-Z_{s})\;\;\;a_{36}=\frac{\partial d}{\partial \kappa }=0\\ a_{35}=\frac{\partial d}{\partial \phi }=-\cos\phi (X-X_{s})-\sin\omega \sin\phi (Y-Y_{s})+\cos\omega \sin\phi (Z-Z_{s})\\ a_{37}=-a_{31}=\frac{\partial d}{\partial X}=-\sin\phi \;\;\;a_{38}=-a_{32}=\frac{\partial d}{\partial Y}=\sin\omega \cos\phi \;\;\;a_{39}=-a_{33}=\frac{\partial d}{\partial Z}=-\cos\omega \cos\phi \end{array}\right.$$

Rewrite Equation (10) into a matrix form:

(12) V=AX-L,P

where:

$$\left\{ \begin{array}{l} V={\left[v_{x},v_{y},v_{d}\right]}^{T}\\ L={\left[l_{x},l_{y},l_{d}\right]}^{T}\\ A=\left(\begin{array}{ccc} a_{11} & a_{12} & a_{13}\\ a_{21} & a_{22} & a_{23}\\ a_{31} & a_{32} & a_{33} \end{array}\;\begin{array}{ccc} a_{14} & a_{15} & a_{16}\\ a_{24} & a_{25} & a_{26}\\ a_{34} & a_{35} & a_{36} \end{array}\;\vdots \;\begin{array}{ccc} -a_{11} & -a_{12} & -a_{13}\\ -a_{21} & -a_{22} & -a_{23}\\ -a_{31} & -a_{32} & -a_{33} \end{array}\right)\\ X={\left[\Delta X_{s},\Delta Y_{s},\Delta Z_{s},\Delta \omega ,\Delta \phi ,\Delta \kappa ,\Delta X,\Delta Y,\Delta Z\right]}^{T} \end{array}\right.$$

**P** is the weight matrix of the image plane coordinates and the depth measurement values. The weight of the measurement data is inversely proportional to the variance of its measurement accuracy. The measurement accuracy of the image plane coordinates depends on the matching accuracy of the SIFT keypoints which is up to sub-pixel level (0.3 pixel is taken in this research). The depth measurement accuracy can be computed by the equation given by Smisek et al. [12] and Daniel et al. [13], so the weight matrix **P** for Kinect V1 is defined as:

(13) $$P=\left(\begin{array}{ccc} \frac{1}{0.3^{2}} & 0 & 0\\ 0 & \frac{1}{0.3^{2}} & 0\\ 0 & 0 & \frac{1}{{\left(2.73\times d^{2}+0.74\times d-0.58\right)}^{2}} \end{array}\right)$$

Through the above steps, the error model of RGB-D camera is built and the camera pose of each frame can be refined by least squares solution of Equation (12). In order to ensure the efficiency while maintaining the precision, bundle adjustment of the image network is realized through a sliding window of five frames.

## 3. Experimental Results

**Figure 5:**
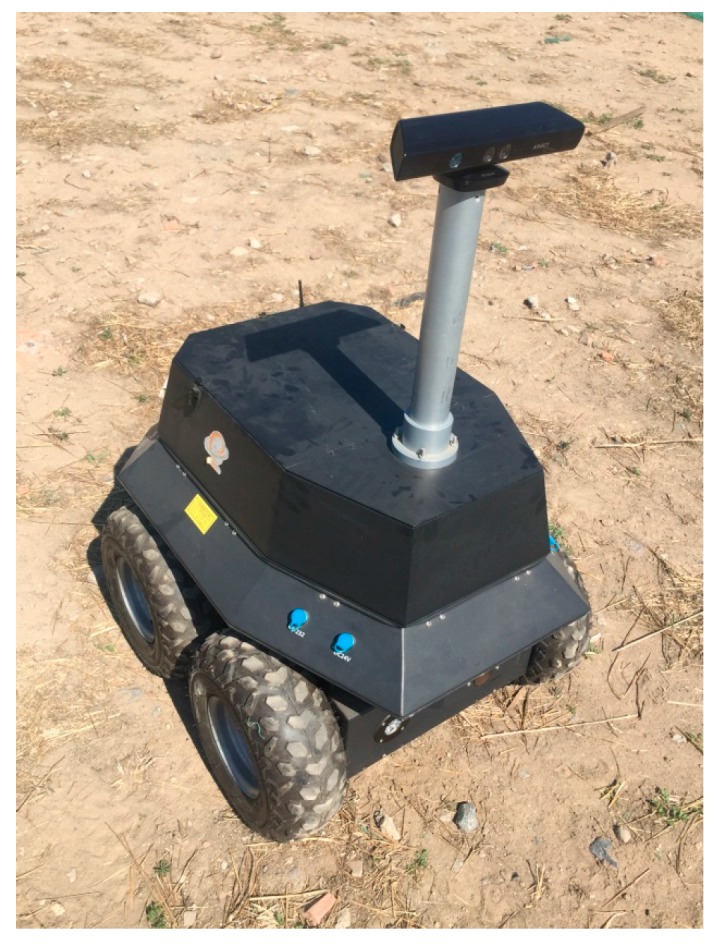
The RGB-D camera mounted on the moving platform.

To verify the actual performance of the proposed method, two field experiments and a contrast experiment using an open RGB-D dataset have been performed. Figure 5 shows the moving platform (model rover) used in these experiments. A Microsoft Kinect V1 camera which has an image resolution of 640 × 480 pixels and a horizontal field of view of 42 degrees was rigidly attached on the top of the camera mast. The camera is about 100 cm above the ground.

Experiment І was carried out in a straight tunnel covering a total distance of approximately 100 m. Several control points were set up as ground truth along the tunnel for accuracy evaluation. Experiment II was performed in an outdoor field to simulate the Lunar and Martian surface. The rover traveled along a loop path with the origin set at [0,0]. The same image was used for the first and last positions to ensure that the true last camera pose was exactly the same as where the first image was recorded. Given that the loop is closed, we can use it to evaluate the accuracy. As the model rover travelled, the RGB-D camera captured RGB frames and depth frames at a rate of 30 fps, while the computer on the rover stored and processed the frames at a rate of 2 fps for SLAM. Considering that the accuracy of the depth camera decreases with the distance increases, depth data within the range of 0.5 m to 4 m were used and depth data outside this range were marked as invalid.

**Figure 6:**
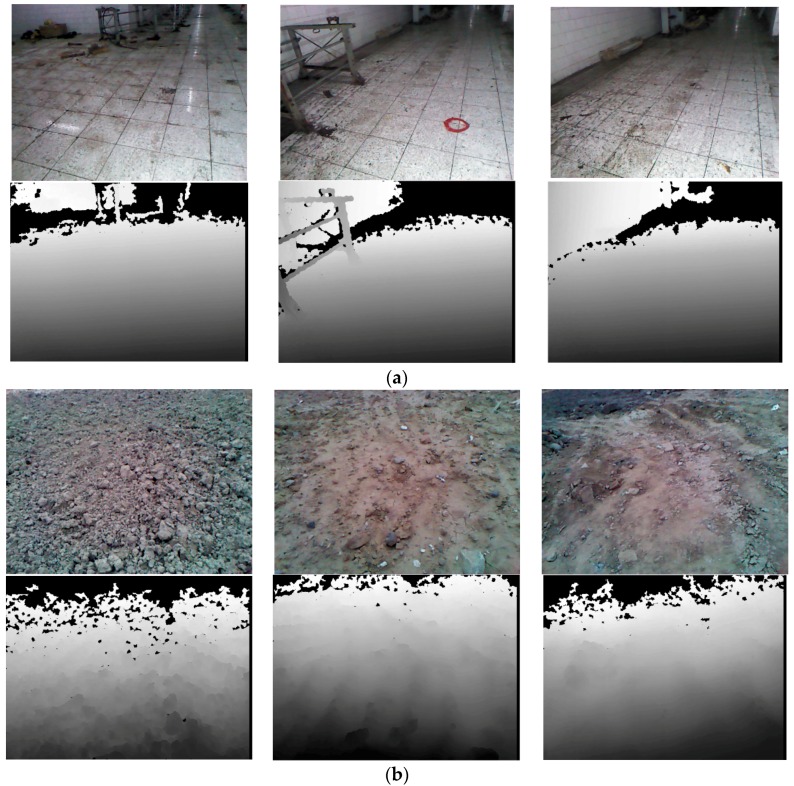
Typical RGB images and corresponding depth images acquired in two experiments. (**a**) Typical images acquired in a tunnel in Experiment I; (**b**) Typical images acquired in an outdoor field in Experiment II. Images in the first row were captured by the RGB camera, and images in the second row were captured by the depth camera. The areas, which are out of the imaging range (0.5 m to 4 m) or without reflected infrared light, are shown in black in the depth images. The middle image of the first row in (a) shows one of the control points (inside the red circle).

In experiment I, the remote-controlled rover travelled from the first control point to the second control point (the distance is 46.97 m) and captured approximately 800 frames of RGB and depth images. In experiment II, the rover traveled about 200 m and captured 2600 frames. Figure 6 shows some typical images acquired by the RGB camera and the depth camera.

The result of our method is compared with the result of the traditional bundle adjustment method. The projection model of the traditional BA method only used the first two equations in Equation (8). The depth information is only used to get the 3D coordinates of the image tie points in the traditional BA. In other words, depth value (*d*) is not considered as observation in the traditional BA; while in the extended BA, the depth measurements are treated as observations and integrated with the image measurements. This is the essential difference between our method and the traditional BA method.

**Figure 7:**
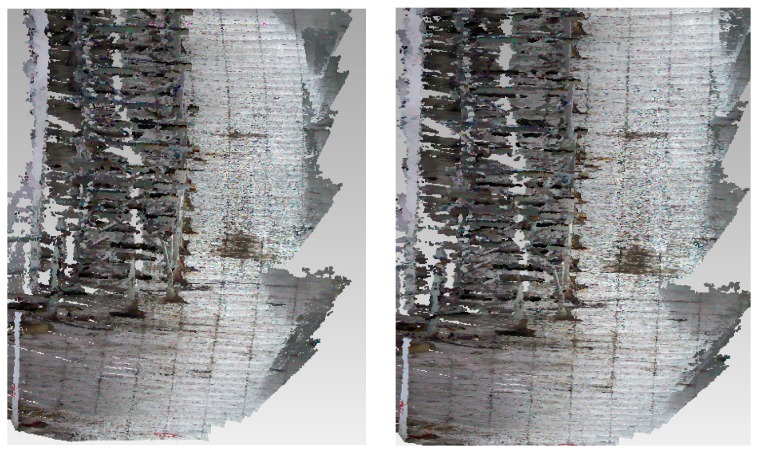
Overhead views of the 3D mapping results in Experiment I. The left figure is the result of the traditional method. The right is the result of the proposed method in this paper.

In experiment I, the localization error of the proposed method is 2.45%, which is notably lower than the 4.22% error of the traditional method. Table 3 shows the statistical results and Figure 7 shows the 3D mapping results.

The details shown in Figure 7 illustrate that the reconstructed scene of our method has smaller deformation. This is because the depth value is brought into the BA model in our method, which makes the BA model take full advantage of the measurement capability of the RGB-D camera. Due to lacking constraint of the depth measurement data, the traditional BA model can only correct the visual measurement error. The steady accumulation of the depth measurement error will cause low accuracy of the position estimation.

**Figure 8:**
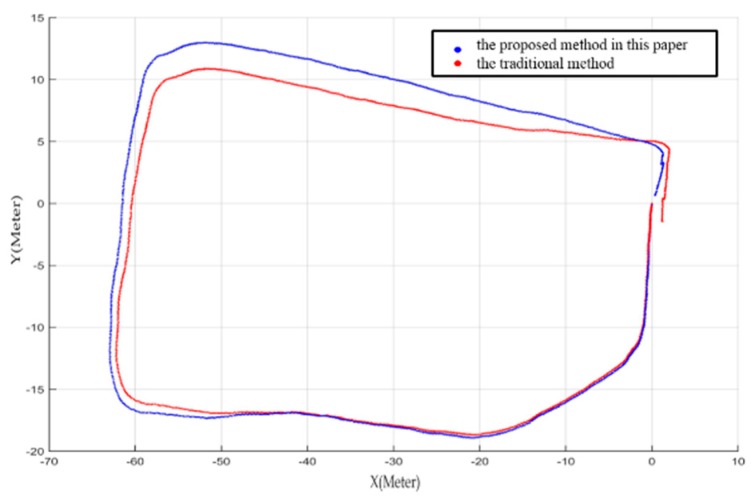
Estimated rover paths from the two BA methods. Red and blue curves represent the estimated trajectory using the traditional method and the proposed method, respectively.

**Figure 9:**
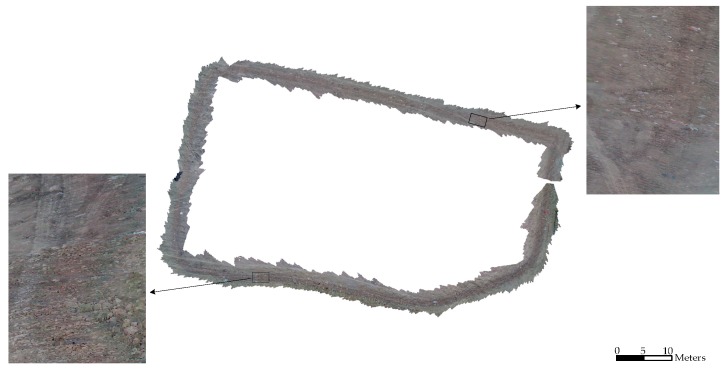
Overhead view of the mapping result of the whole scene in experiment II using our method. The two insets are detailed 3D views of the areas in the two rectangles.

In Experiment II, the error of the proposed method is 2.48%, which is better than the 3.84% error obtained by the traditional method. The statistical result is shown in Table 4 and the estimated rover paths are shown in Figure 8 and Figure 9.

From Figure 8, obvious improvement can be seen with the closure error decreases from 3.84% to 2.48% using the proposed method in this paper. The mapping result in Figure 9 shows that our method can obtain a highly accurate map even without loop closure detection. Each inset in the Figure covers an area of about 3 m in length and consists of about 40 frames of point clouds. The detailed maps show that there are no gaps or artifacts between each frame, meanwhile objects on the ground such as small rocks and the tracks of the rover are reconstructed seamlessly in high precision.

To compare with the methods mentioned in [22,23] which use depth values as a constraint condition in BA model, another contrast experiment is preformed to verify the performance of the proposed method using an open RGB-D dataset which is available at http://vision.in.tum.de/data/ datasets/rgbd-dataset. The dataset is obtained in an indoor environment by Kinect V1. Three datasets (fr1/xyz, fr1/desk, fr3/room) with long and complex trajectories are selected in this experiment. The average of the RMSE values of the absolute trajectory error (ATE) of our method is 0.08 m. In contrast, the average of the ATE RMSE values for frame-to-frame tracking without loop closure detection is 0.19 m and the ATE RMSE values for frame-to-keyframe tacking with loop closure detection is 0.07 m [22]. From the experiment result, we can see that our method is more accurate than the compared method without loop closure detection and reaches the same level of the compared method with loop closure detection. Numerous studies show that loop closure can effectively improve the pose estimation accuracy. So our BA model takes full advantage of the measurement capability of the RGB-D camera system and provides improved performance of SLAM for open loop route, which is particularly applicable for planetary rover localization and mapping.

It should be noted that the developed BA model in this paper is based on Microsoft Kinect V1. The camera model (i.e., Equation (8)) is also applicable to Kinect V2. We have also experimented using Kinect V2 by changing the weight matrix using the error characteristics of the depth camera of Kinect V2 [14]. Due to the improvements of image resolution and depth measurement accuracy, the localization error is slightly better than that of Kinect V1. As a typical example, for an outdoor route of 88.9 m, the localization closure error is 2.03%.

## 4. Conclusions

In this paper, we presented an extended BA-based SLAM method using a RGB-D camera to decrease the drift and refine the camera pose parameters for motion estimation. We concentrated on verifying the localization and mapping ability of RGB-D camera onboard a rover that could be used in a GPS-denied environment such as lunar and Martian surface. 2D and 3D feature points extracted from visual RGB images and 3D point clouds were used as tie points between consecutive frames. Based on the characteristics of the RGB-D camera, a new projection model of RGB-D camera was built using both types of primary measurement data (the image plane coordinates and the depth values). Moreover, we built an accurate error model based on the projection model. The new BA model was applied to the image network with a sliding window to gain accurate pose estimation results efficiently. Field experiment results demonstrated that the proposed method notably improves localization accuracy when compared with the traditional method.

Our method provides an effective way to build an accurate geometric model of a RGB-D camera. The developed BA model is suitable for Microsoft Kinect V1 and V2. When it is applied to other RGB-D sensors, the model may need to be modified, especially for the weight matrix in the error model.

## Figures and Tables

**Table 1:** Internal parameters of depth camera and RGB camera. The unit for *f_x_*, *f_y_*, *x*_0_, *y*_0_ is pixel.

	*f_x_*	*f_y_*	*x*_0_	*y*_0_	*k*_1_	*k*_2_	*k*_3_	*p*_1_	*p*_2_
Depth	519.95	519.55	315.82	238.71	0.04810	0.19281	0.0	0.00458	0.00014
RGB	584.35	584.33	317.97	252.80	0.10585	0.27096	0.0	0.00504	0.00166

**Table 2:** External parameters of depth camera and RGB camera.

**Rotation Angles (degree)**	−0.00079	−0.00084	−0.00541
**Translation Vector (mm)**	−25.59983	0.16700	−0.40571

**Table 3:** Localization results of experiment I with a ground truth length of 46.97 m.

	Calculated Length (m)	Error
Our method	45.82	2.45%
Traditional method	44.99	4.22%

**Table 4:** Localization results of experiment II with a total length of 183.5 m.

	Closure Error(m)	Error
Our method	4.56	2.48%
Traditional method	7.05	3.84%
